# Dysmenorrhea and Premenstrual Syndrome in Association with Health Habits in the Mexican Population: A Cross-Sectional Study

**DOI:** 10.3390/healthcare12212174

**Published:** 2024-10-31

**Authors:** Julia María Alatorre-Cruz, Graciela Catalina Alatorre-Cruz, Vianey Marín-Cevada, Ricardo Carreño-López

**Affiliations:** 1 Centro de Investigación en Ciencias Microbiológicas, Instituto de Ciencias, Benemérita Universidad Autónoma de Puebla, Puebla 72575, Mexico; fcb.colaborador06@correo.buap.mx (J.M.A.-C.); vianey.marin@correo.buap.mx (V.M.-C.); 2 Unidad de Investigación en Neurodesarrollo, Instituto de Neurobiología, Universidad Nacional Autónoma de México, Querétaro 76230, Mexico; galatorrec@comunidad.unam.mx

**Keywords:** eating habits, physical activity, sleep habits, dysmenorrhea, premenstrual syndrome

## Abstract

Background: Dysmenorrhea and premenstrual syndrome (PMS) are common disorders in the Mexican population, but these are usually underdiagnosed and under-treated, impacting women’s quality of life. Adequate health habits have been reported as precursors of decreasing dysmenorrhea symptoms. However, few studies assess their impact on PMS. Aim: This study aims to evaluate dysmenorrhea and premenstrual syndrome in association with health habits in the Mexican population. Methods: To assess the impact of health habits on menstruation symptoms a validated survey was conducted in 1679 adult females aged ≥18 years. The survey collected data on participants’ dysmenorrhea, PMS, and their health habits. Results: The analysis showed that physical activity duration, changes in eating habits (increases in salty or sugary foods) during menstruation, and oversleeping habits predict increases in dysmenorrhea and PMS. In contrast, an active sexual life, relaxing physical activity, and adequate sleep hours during menstruation seem to decrease the symptoms. Conclusions: We conclude that adequate health habits and addressing early gynecological conditions might regulate dysmenorrhea and PMS.

## 1. Introduction

Dysmenorrhea is the most common gynecological disorder in reproductive age and is becoming a health problem worldwide [1] because the condition leads to absence from work or academic activities, impairing women’s quality of life and their life plans. Despite its common occurrence, dysmenorrhea is underdiagnosed and under-treated [2]. This disorder entails recurrent pain, with characteristics of deregulated somatosensory processing of the central nervous system CNS [3], severe abdominal spasms and cramps, heavy menstrual flow, nulliparity, anxiety, depression, problems in sexual functioning [4,5], headaches, backaches, dizziness, irritability, breast tenderness, swelling of extremities, diarrhea, and nausea [6].

The symptoms and signs differ for each woman; previous studies have classified these pain symptoms between 0 and 3, with 3 representing the most severe pain [7], characterized by non-responsiveness to analgesics. Moreover, the severity of symptoms of dysmenorrhea positively correlated with the age of menarche, the regularity of the menstrual period, and the duration and amount of menstrual flow [4,8].

There are two types of dysmenorrhea: primary and secondary. Primary dysmenorrhea or pain during menstruation, in the absence of pelvic pathology, is a common disease characterized by pain before and after the menstrual period; this dysmenorrhea has been reported as genetically influenced, while secondary dysmenorrhea entails a pathological condition with an anatomical problem, for example, endometriosis. The pain typically lasts from 8 to 72 h, with most severity on the 1st and 2nd days of menstruation because of the increased release of prostaglandins during this period [9]. In general, menstrual symptoms have been classified into menstrual-related diagnoses such as dysmenorrhea or PMS. Dysmenorrhea is more related to the symptoms of pain accompanying menses, while PMS is generally related to emotional or psychological concerns [4].

On the other hand, inappropriate dietary habits, low physical activity, and poor sleeping habits have been reported as precursors of menstrual disorders [1,10]. Interestingly, prior studies reported that breakfast or meal skipping predicts female gynecologic disorders [7,10]. Additionally, multiple studies reported that certain nutrient intake, such as fatty acids, antioxidants, the combination of vitamins and minerals [11], consumption of fruits, vegetables, fish, milk, and dairy products, low amounts of consumed salt [7], low frequency of drinking coffee [8], the Mediterranean diet [6], and a lower amount of consumed kilocalories [12], positively affects dysmenorrhea symptoms [6,7,8,10,11,12].

Prior studies have associated high-level physical activity with a reduction in dysmenorrhea severity [12]. They explain this association as physical activity decreasing levels of C-reactive protein (HsCRP), PGE2, and PGF2α, resulting in the amelioration of uterine contraction and inflammation [12,13]. Previous studies in the Mexican population also reported that relaxation exercises induced by physiotherapy seem to decrease symptoms of primary dysmenorrhea [14], while in other populations, relaxation exercises improve menstrual pain and quality of life [15,16,17,18].

Multiple studies reviewed the relationship between sleep and menstrual disturbances. PMS and dysmenorrhea were associated with sleep disturbances such as sleep quality, daytime sleepiness, difficulty initiating/maintaining sleep, and duration. Abnormal menstrual cycles and heavy bleeding were related to sleep quality and difficulty initiating/maintaining sleep [19], while sleeping less than five hours increased PMS [20].

In Mexico, the prevalence of dysmenorrhea is high, and 62.4% of women suffer severe pain, as measured by the Visual Analog Scale (VAS) [21]. Moreover, a high percentage of the Mexican population does not have access to formal medical care or pharmacologic treatment [21]. Therefore, women use strategies of self-medication and alternative therapies to respond to dysmenorrhea symptoms [22]. However, for between 28% and 90% of Mexican women, the pain is often not relieved despite the use of medication [21,23,24,25,26]. Therefore, the focus on health habits is a useful approach for understanding how non-medical interventions could play a role in managing dysmenorrhea symptoms.

Recent studies report that Mexican women show an inability to participate in daily activities (1–6 menstrual cycles per year), require incapacitation (6–24 h per cycle), and have school absenteeism (1–13 menstrual cycles per year) [23]. Additionally, another study suggests that menstrual pain affects Mexican women´s academic achievement due to absenteeism [27].

Although there are no previous studies assessing dysmenorrhea effect on the Mexican economy, previous studies in the US reported that absenteeism due to dysmenorrhea symptoms generates 600 million work hours resulting in a loss of 2 billion USD per year. We suggest a similar situation in the Mexican economy.

Additionally, between 78% and 90% of the Mexican population prefers sweetie beverages and fast food with high amounts of saturated fat and added sugar. Moreover, their legumes, fruits, and vegetables consumption is low, between 6.3 and 8.2% lower than that recommended by the Food and Agriculture Organization (FAO) [28]. Therefore, this exploratory study pretends to analyze retrospectively whether dietary habits, physical activity, and sleeping habits affect dysmenorrhea symptoms and PMS. We would expect more acute dysmenorrhea symptoms and more severe PMS associated with inappropriate health habits.

## 2. Material and Methods

### 2.1. Participants

This cross-sectional study was conducted on 1679 females (See Table 1), all of them were enrolled online from 2022 to 2023 (statistical power = 0.79). They were ethnic Mexican and native Spanish speakers and had at least six years of education. We included participants who completed the survey and signed the consent for participation. We excluded from our cohort those who reported suffering from endometriosis or cancer. All volunteers were informed of their rights and provided written informed consent for participation in this study. This research was carried out ethically and was approved by the Benemérita Universidad Autónoma de Puebla.

### 2.2. Procedure

The data were obtained from a self-administered survey directed at females over 18 years, who reported at least their first menarche. The survey assesses participants’ dysmenorrhea symptoms, PMS, and health habits. This survey entails one hundred and twenty-two items distributed in five sections: (1) identification data; (2) anthropometric data; (3) symptoms of dysmenorrhea and PMS index (See Table 2); (4) health habits: dietary habits, physical activity, and sleep habits; and (5) Other habits (See Table 2).

### 2.3. Validation

The statistical analysis for the survey’s validation was performed using a chi-square test. The factor analysis technique assessed the items with an orthogonal rotation, “Varimax”. In this analysis, the factor weight of each item was at least 0.4. We also measured the internal consistency of each item for each factor, exploring their reliability using Cronbach’s alpha (0.724). The participants reported being comfortable with all items and completion times and responded 100% to the survey (See survey in Appendix A).

### 2.4. Data Analysis Methods

#### 2.4.1. Distribution Analysis of Health Habits

We compared the symptoms of dysmenorrhea and PMS index between participants with different dietary habits (adequate (AD) versus inadequate (ID)), physical activity (passive (PA) versus active (AA) during menstruation), and sleep habits (less sleep (<6 h) (LS) versus more sleep (>6 h) (MS) during menstruation) using independent t-tests.

PA was defined as unstructured daily activities, such as working, housekeeping, transportation, and leisure, while AA involved structured activities that change the capacity of the cardiorespiratory system [29].

For eating habits, a K-means clustering was performed to determine the quality of participants’ nutritional habits based on a prior Mexican study [30]. The variables included were intake of vegetables, fruits, cereals, meat, fish, dairy products, oilseeds, fats, eggs, snacks, soda, and coffee, eating habits during the menstrual period, and type of favorite foods during menstruation. The clustering analyses resulted in 827 participants in the ID group and 852 in the AD group.

Additionally, we compared the distribution of favorite food types during menstruation (salty, sweet, bitter, none in particular) with the severity of dysmenorrhea and PMS symptoms using a chi-square test. We also performed a K-means clustering analysis to classify participants into two groups based on symptom severity: more menstrual symptoms (MMSs) and fewer menstrual symptoms (LMSs). The clustering analysis resulted in 771 participants classified as having MMSs and 906 participants classified as having LMSs.

#### 2.4.2. Predictors of Menstruation Symptoms

Regression analyses were performed to identify the association between dysmenorrhea and the PMS index and those factors that might explain the variability in menstrual symptoms. The linear regression included the index as the dependent variable and age, sex, scholarship, anthropometric data, dietary and sleep habits, physical activity, substance abuse, and sexual habits as independent variables. The linear regression analyses included multiple-linear backward regressions to find a reduced model that best explains the data.

## 3. Results

### 3.1. Distribution of Health Habits

**Figure 1:**
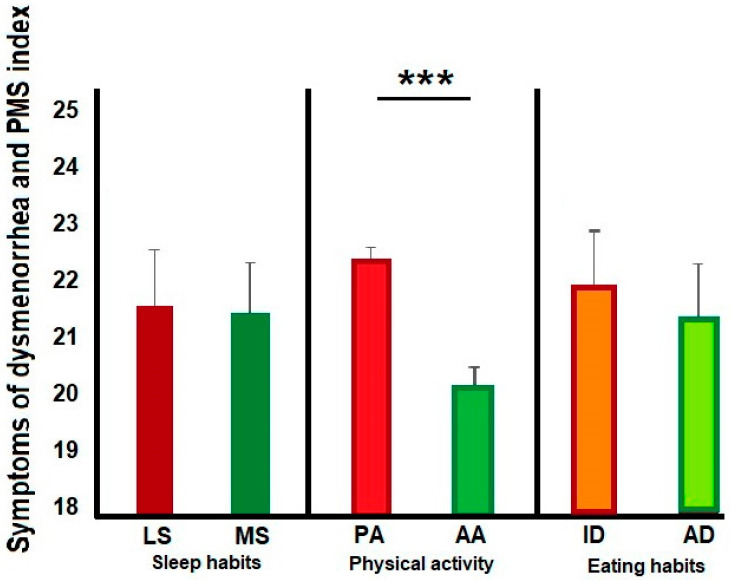
The bar graphs show differences in this cohort’s health habits distribution. We found that the participants with active physical activity (AA) exhibited a lower index score than those with passive physical activity (PA). No differences were observed in sleep and eating habits. *** Significant difference *p* < 0.0001.

As we expected, physical activity (PA versus AA) was associated with menstrual symptoms (t(1676) 6.6, *p* < 0.0001). The PA group had a greater score in the index than the AA (PA, mean (M) = 22.7, SD = ±8.5; AA, M = 20.0, SD = ±7.9) during menstruation. Although menstrual symptoms did not differ between sleep and eating habits (sleep habits, LS vs. MS; t(1676) 0.2, *p* = 0.85; LS, M = 21.8, SD = ±8.7; MS, M = 21.7, SD = ±8.3; Eating habits ID vs. AD, t(1676) −1.6, *p* = 0.11; ID, M = 22.0, SD = ±8.5; AD, M = 21.4, SD = ±8.2). (See Figure 1). We found differences between the MMS and LMS groups in the type of favorite foods during menstruation (X2 (3) 45.1, *p* < 0.0001), with a higher count than expected for the MMS group in sweet and salty flavors compared to the LMS group.

### 3.2. Predictors of Menstrual Symptoms

**Figure 2:**
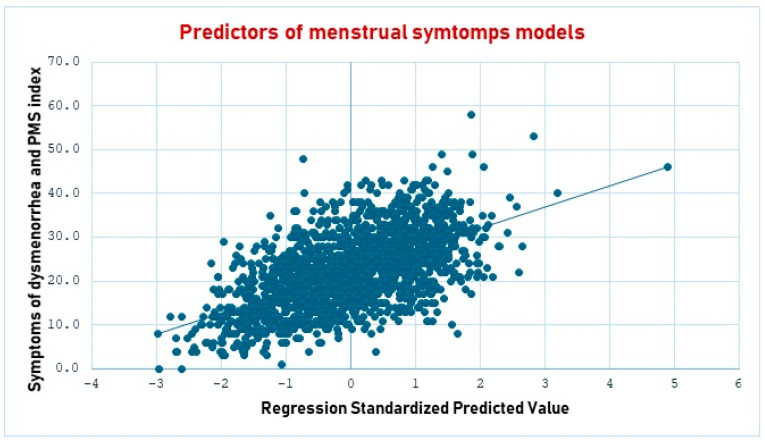
The scatter plot shows the regression standardized predictive value of the statistical model to predict menstrual symptoms. The line in blue represents the expected values in the regression model.

As shown in Table 3, menstrual diseases, the duration of the menstrual cycle, bleeding, pain, physical activity, change of eating habits, usual sleep duration, and frequency of vegetable intake were predictors of higher scores in the symptoms of dysmenorrhea and PMS index. In contrast, age, sex life, physical activity, and sleep hours during menstruation predicted lower scores in the symptoms of dysmenorrhea and PMS index. (See Figure 2).

## 4. Discussion

This study aimed to determine whether dietary habits, physical activity, and sleeping habits affect dysmenorrhea and PMS symptoms. As expected, participants with inappropriate health habits had higher menstrual symptoms. However, some unexpected results warrant further explanation.

### 4.1. Sociodemographic, Anthropometric, and Characteristics of Menstrual Cycle as Predictors

Although in our sample, age was an essential predictor for suffering from dysmenorrhea and PMS, previous studies reported that this variable depended on other factors such as earlier menarche (younger than 12 years), longer menstrual cycles, heavy menstrual flow, BMI < 20 kg/m^2^, and smoking habit [31,32]. This interaction between age and other factors was also observed in our sample, where most volunteers reported their first menarche between ages 11 and 13.

In our study, early menarche was associated with higher menstrual symptoms, which match multiple studies reporting that earlier menarche triggers primary dysmenorrhea; these studies also suggest that this condition is associated with hormonal immaturity and higher exposition to prostaglandins F2α (PGF2α), disturbing smooth muscle contractions of the uterus, emotional factors, and the reproductive system, increasing the chance of higher pain during menstrual cycle [5,33,34,35,36].

Recent studies also describe that longer menstrual cycles and heavy menstrual flow were good predictors of menstrual symptoms [4,8,31,32], which matches our findings.

Given that our sample represents the Mexican population, most of the participants suffered from obesity, and BMI was not a predictor variable because of its poor variability in our sample. Furthermore, smoking was not a predictor of menstrual symptoms, likely because only 10.3% of participants reported this habit.

In our findings, we also observed that gynecological medical records from the participants were associated with the severity of menstrual symptoms. Prior studies also matched our findings. They reported that dysmenorrhea is genetically influenced [3] and positively associated with its severity [31].

Interestingly, active sexual life was a significant predictor of fewer menstrual symptoms in our study, although we did not find previous research supporting this finding. We suppose that sexual life is a delicate topic worldwide, which affects the participant’s willingness to respond about their sexual habits. In Mexico, public information on female sexual activity is generally available only after the first pregnancy [37], leaving a gap in understanding sexual behavior during childhood or adolescence.

In our study, the number of days of pain during menstruation was a strong predictor of more severe menstrual symptoms. Previous studies reported that the pain appears when the female suffers from dysregulated hormonal equilibrium because of high estriol levels [38], which is also associated with uterine contraction and inflammation. Moreover, it has been reported that C-reactive protein (HsCRP), PGE2, and PGF2α levels are also involved in this inflammation process [12,13]. Therefore, the results observed in this study align with the established pathophysiology of menstrual pain.

### 4.2. Physical Activity Predictors

As anticipated, physical activity is associated with lower dysmenorrhea symptoms, mainly when females practice it during menstruation with a short duration. These results match prior reports describing that physical activity during menstruation reduces the incidence of dysmenorrhea [12] and increases the quality of life when the woman practices pilates [39] or relaxation exercises, such as yoga [14,15,16,17,18,40]. Other studies explain that physical activity decreases C-reactive protein (HsCRP), PGE2, and PGF2α levels, ameliorating uterine contraction and inflammation [12,13]. However, no studies explain the effect of physical activity on PMS, even when premenstrual symptoms, like anger, anxiety, depression, activity level, fatigue, and menstrual distress symptoms, such as cramps, aches, and swelling, are modulated by the same hormones: estradiol, prolactin, progesterone, and cortisol [13]. Therefore, our study contributes to the literature by demonstrating that physical activity positively affects PMS as well.

### 4.3. Eating Habits Predictors

We expected adequate eating habits would be associated with lower menstrual symptoms, and our findings partially matched our expectations. We found that an increased intake of sugary (55.3%) or salty (19.5%) foods during the menstrual period was associated with higher menstrual symptoms, as previous studies reported healthier food habits were not a good predictor [7,31,41]. However, no specific food influences menstrual symptoms; we suggest that we did not find any food intake as a predictor of dysmenorrhea symptoms because we did not have precise information about eating consumption during the menstrual cycle.

### 4.4. Sleep Habits Predictors

We expected adequate sleep habits would be associated with lower menstrual symptoms; our findings matched our expectations. We found that inadequate sleep hours (oversleeping) were associated with higher menstrual symptoms. As mentioned before, sleep disturbance is associated with higher menstrual symptoms [19,20]. Even when we did not assess sleep disturbances in our sample, our participants who tend to sleep too many hours may suffer from sleep disturbances. For example, polycystic ovary syndrome has been associated with an increased risk for sleep-disordered breathing [42], or circadian cycle dysregulation may generate an increase in morbidity risk for inflammatory diseases such as cardiovascular disorder [43,44]. Sleep disturbances mainly affect chemical substances produced during inflammation, such as energy-related molecules, nitric oxide, cytokines, and prostaglandins [45]. Therefore, our participants with habitual oversleeping (not during menstruation) might have more inflammatory symptoms and pain during menstruation than the remaining. In addition, we found that more sleep during menstruation was related to decreased menstrual symptoms, as suggested by previous studies [31].

## 5. Conclusions

Despite the limitations derived from the study design, we concluded that eating habits predict higher scores in the symptoms of dysmenorrhea and PMS. Sleep hours during menstruation were associated with dysmenorrhea and PMS and regular physical activity was a critical factor in managing dysmenorrhea and PMS.

## 6. Limitations

The present study has inherent limitations. It is cross-sectional, meaning that it may not provide sufficient evidence to make conclusions on predictors of menstrual symptoms. In addition, given that no physician examined our participants, they might suffer from other psychological and medical conditions. Additionally, we did not assess the 24 h dietary recall method during the menstrual cycle, the type or intensity of physical activity, fatigue, diurnal sleepiness, or sleep disorders. Therefore, our study’s interpretations should be carried out carefully.

## Figures and Tables

**Table 1:** Demographic and anthropometric data.

Variable	Mean (±SD)
Age (years)	30.4 (±7.8)
	n (%)
Scholarship (years of education)	6 years, 119 (±7.1)
	9 years, 28 (±1.7)
	12 years, 114 (±6.8)
	16 years, 657 (±39.1)
	18 years, 761 (45.3)
	Mean (±SD)
Height (cm)	160 (±0.6)
Weight (kg)	65.6 (±14.3)
BMI	25.4 (±5.2)
Age of first menarche (years)	12.1 (±1.6)

Note: SD = standard deviation; BMI = body mass index.

**Table 2:** Symptoms of dysmenorrhea and PMS index.

Items	Responses
Pain at the beginning of the menstruation	0–10 0–null 10-pain intensive
Pain at the end of the menstruation	0–10 0–null 10-pain intensive
Did you have ever suffered from hormonal imbalances?	Yes (1) No (0)
Did you have difficulty getting pregnant?	Yes (1) No (0)
Did you have ever had infertility?	Yes (1) No (0)
Have you had abortions?	Yes (1) No (0)
Did you have ever had fibroids?	Yes (1) No (0)
Did you have ever had polycystic ovary syndrome disease?	Yes (1) No (0)
Have you had a hysterectomy?	Yes (1) No (0)
Did you have ever had dizziness?	Never, rarely, frequently, always
Did you have ever had vomiting?	Never, rarely, frequently, always
Did you have ever had nausea?	Never, rarely, frequently, always
Did you have ever had diarrhea?	Never, rarely, frequently, always
Did you have ever had constipation?	Never, rarely, frequently, always
Did you have ever had fainting?	Never, rarely, frequently, always
Have you had a headache?	Never, rarely, frequently, always
Have you had chills?	Never, rarely, frequently, always
Have you had abdominal inflammation?	Never, rarely, frequently, always
Have you had edema or inflammation in your legs?	Never, rarely, frequently, always
Did you experience sadness?	Yes (1) No (0)
Did you experience anger or annoyance?	Yes (1) No (0)
Did you experience anxiety?	Yes (1) No (0)
Did you experience aggressiveness?	Yes (1) No (0)

**Table 3:** Regression model: predictors of menstrual symptoms.

Independent Variable	Predictor Variables	Standardized Coefficients			ANOVA		
		Β	T	*p*-Value	R^2^	F	*p*-Value
Symptoms of dysmenorrhea and PMS index		18.03	10.38	<0.0001	0.45	33.6	<0.0001
	Age	−0.138	−6.008	<0.0001			
	Menstrual diseases	0.145	6.28	<0.0001			
	Sex life	−0.052	−2.310	0.021			
	Menstrual cycle (days)	0.049	2.146	0.032			
	Duration of menstrual bleeding (days)	0.146	6.659	<0.0001			
	Pain (dp)	0.112	4.964	<0.0001			
	Physical activity (dp)	−0.187	−6.941	<0.0001			
	Physical activity duration (dp)	0.063	2.349	0.019			
	Change of eating habits (dp)	0.235	10.390	<0.0001			
	Sleep hours	0.079	2.921	0.004			
	Sleep hours (dp)	−0.114	−4.180	<0.0001			

Notes: dp = during menstrual period.

## Data Availability

The data presented in this study are available upon request from the corresponding author. The data are not publicly available because they contain sensitive patient information.

## Appendix A

Table A1 illustrates the survey of participants’ dysmenorrhea symptoms, PMS, and health habits. We included all items applied to our participants.

**Table A1:** Survey of participants’ dysmenorrhea symptoms, PMS, and health habits.

Identification Data (Datos de Identificación)	Possible Responses
Age/(Edad)	Years (años)
Marital status/(Estado civil)	Single/Married/Common union/Divorced/Widowed (Soltero (a)/Casado (a)/Unión libre (a)/Divorciado (a)/Viudo (a))
Occupation/(Ocupación)	Open (Abierta)
Education /(Escolaridad)	None/Primary/Secondary/High School/Undergraduate/Graduate (Ninguno/Primaria/Secundaria/Preparatoria/Licenciatura/Posgrado)
Residencie’ place/(Lugar de residencia)	Open (Abierta)
Nationality/(Nacionalidad)	Open (Abierta)
Anthropometric data (Datos antropométricos)	
Height(m)/(Estatura)	Meters (mts)
Weight (kg)/(Peso)	kilograms (kg)
Medical records (Datos clínicos)	
Pathological family history (Antecedentes familiares patológicos)	
Did your family have ever had Dysmenorrhea?/ (En tu familia, han padecido dismenorrea?)	Yes/No (Sí/No)
Did your family have ever had difficulty getting pregnant?/(En tu familia, han tenido dificultad para embarazarse?)	Yes/No (Sí/No)
Did your family have ever had fibroids?/(En tu familia, han padecido miomas (tumores beningnos)?)	Yes/No (Sí/No)
Did your family have ever had cancer?/(En tu familia, han padecido cancer?)	Yes/No (Sí/No)
Did your family have ever had endometriosis?/(En tu familia, han padecido endometriosos?)	Yes/No (Sí/No)
Did you family have ever had a hysterectomy?/(En tu familia, le han hecho histerectomía?)	Yes/No (Sí/No)
Pathological personal history (Antecedentes personales patológicos)	
Did you have ever had polycystic ovary syndrome disease?/(¿Usted padece Sindrome de Ovario Poliquístico?)	Yes/No/I have not checked myself (Si/No/No me he checado)
Did you have ever had diabetes?/(¿Usted padece Diabetes?)	Yes/No/I have not checked myself (Si/No/No me he checado)
Did you have ever had hypertension (high blood pressure)?/(¿Usted padece Hipertensión (presión alta)?)	Yes/No/I have not checked myself (Si/No/No me he checado)
Did you have ever had anemia?/(¿Usted padece anemia?)	Yes/No/I have not checked myself (Si/No/No me he checado)
Did you have ever had Autoimmune diseases (arthritis, ankylosing spondylitis, lupus erythematosus, antiphospholipid syndrome)?/Usted, padece enfermedades autoinmunes (artritis, espondilitis anquilosante, lupus eritematoso, Síndrome antifosfolípidos)?	Yes/No/I have not checked myself (Si/No/No me he checado)
Did you have ever had cholesterolemia?/(¿Usted padece hipercolesterolemia o colesterol alto?)	Yes/No/I have not checked myself (Si/No/No me he checado)
Did you have ever had hypertriglyceridemia?/(¿Usted padece hipertrigliceridemia o triglicéridos altos?)	Yes/No/I have not checked myself (Si/No/No me he checado)
Did you have ever had difficulty getting pregnant?/(Usted, ha tenido dificultad para embarazarse?)	Yes /No (Sí/No)
Did you have ever suffered from infertility?/(Usted, ha sufrido de infertilidad?)	Yes /No (Sí/No)
Have you ever have had abortions?/(Usted, ha tenido abortos?)	Yes /No (Sí/No)
Did you have ever had endometriosis?/(Usted padece de endometriosis?)	Yes /No (Sí/No)
Did you have ever had fibroids? /(Usted padece de miomas (tumores benignos)?)	Yes /No (Sí/No)
Did you have ever had hormonal imbalances? /(Usted padece de alteraciones hormonales?)	Yes /No (Sí/No)
Did you have ever had cancer? /(Usted padece de cancer?)	Yes /No (Sí/No)
Have you ever had a hysterectomy? /(Usted tuvo una histerectomía (quitar la matriz)?)	Yes /No (Sí/No)
Have you ever had vaginal infecctions? (Usted ha tenido infecciones vaginales?)	Yes /No (Sí/No)
Premenstrual syndrome (Síndrome premenstrual)	
Have you ever had abdominal inflammation?/(En su período menstrual, usted padece dolor abdominal?)	Never, rarely, frequently, always (Nunca, rara vez, frecuentemente, siempre)
Have you ever had chills?/(En su período menstrual, usted tiene escalofríos?)	Never, rarely, frequently, always (Nunca, rara vez, frecuentemente, siempre)
Have you ever had edema or inflammation in your legs?/(En su período menstrual, usted tiene edema o inflamación en sus piernas?)	Never, rarely, frequently, always (Nunca, rara vez, frecuentemente, siempre)
Have you ever had a headache?/(En su período menstrual, usted padece de dolores de cabeza?)	Never, rarely, frequently, always (Nunca, rara vez, frecuentemente, siempre)
Did you experience sadness?/(En su período menstrual, usted experimenta tristeza?)	Never, rarely, frequently, always (Nunca, rara vez, frecuentemente, siempre)
Did you experience anger or annoyance?/(En su período menstrual, usted experimenta enojo?)	Never, rarely, frequently, always (Nunca, rara vez, frecuentemente, siempre)
Did you experience anxiety?/(En su período menstrual, usted experimenta ansiedad?)	Never, rarely, frequently, always (Nunca, rara vez, frecuentemente, siempre)
Did you experience aggressiveness?/(En su período menstrual, usted experimenta agresividad?)	Never, rarely, frequently, always (Nunca, rara vez, frecuentemente, siempre)
Indicate level of bleeding in the first days of menstruation (Indique nivel de sangrado en los primeros días de la menstruación)	No menstruation/Light/Normal/Abundant/Very abundant (No menstruo/Ligero/ Normal/Abundante /Muy abundante)
Indicate level of bleeding in the last days of menstruation. (Indique nivel de sangrado en los últimos días de la menstruación.)	No menstruation/Light/Normal/Abundant/Very abundant (No menstruo/Ligero/ Normal/Abundante /Muy abundante)
Bleeding duration (days): (Duración de sangrado (días):)	0–7 days (días)
Have you stopped working, studying or doing your activities because of colic pain? (¿Has dejado de trabajar, estudiar o hacer tus actividades por el dolor de cólico?)	Yes/No/Sometimes (Si/No/A veces)
From 0 to 10, how much it hurts in the first days of your menstrual period (Del 0 al 10 que tanto te duele en los primeros días en tu período menstrual)	0–10
From 0 to 10, how much it hurts you in the last days of your menstrual period (Del 0 al 10 que tanto te duele en los últimos días en tu período menstrual)	0–10
From 0 to 10, how much anxiety (fear and restlessness) you have in the first days of your menstrual period (Del 0 al 10 que tanta ansiedad (miedo, temor e inquietud) presentas en los primeros días de tu período menstrual)	0–10
From 0 to 10, how much stress (physical or emotional tension) you experience in the first days of your menstrual period (Del 0 al 10 que tanto estrés (tensión física o emocional) presentas en primeros días de tu período menstrual)	0–10
From 0 to 10, how much energy or spirit you have in the first days of your menstrual period (Del 0 al 10 que tanta energía o ánimo presentas en los primeros días de tu período menstrual)	0–10
Do you use medication for colic pain? (¿Usas medicamentos para el dolor de cólico?)	Yes /No (Sí/No)
What medication do you take for colic and how many pills per day? (¿Cuál medicamento tomas para el cólico y cuántas pastillas por día?)	Open (Abierta)
Do you use hormonal treatment or contraceptives? (¿Usas tratamiento hormonal o anticonceptivos?)	Yes/No (Sí/No)
If so, what contraceptive method do you use? (¿De ser así, cual método anticonceptivo usas?)	Open (Abierta)
Gynecobstetric data (Datos gineco-obstétricos)	
How often do you go to the gynecologist? (¿Con que frecuencia acudes a la ginecóloga (o)?)	Never/Once a year/Once every 6 months/More than once every 6 months (Nunca/1 vez al año/1 vez cada 6 meses/Más de 1 vez cada 6 meses)
How often have you had a pap smear? (¿Con que frecuencia te has hecho el papanicolaou?)	Never/Once a year/Once every 6 months/More than once every 6 months (Nunca /1 vez al año/1 vez cada 6 meses/ Más de 1 vez cada 6 meses)
Sex life: (Vida sexual:)	Active/Inactive (Activa/Inactiva)
Age of menarche (Edad de la primera menstruación)	Years (años)
Indicate what is the time of your menstrual cycle (Indicar cual es el tiempo de su ciclo menstrual)	Less than 21 days/From 21–35 days/Greater than 35 days/No Menstruation (Menos de 21 días/ De 21–35 días/Mayor a 35 días /No Menstruo)
Please indicate if you are currently: (Indique si usted está:)	None/Pregnant/Breastfeeding/Menopause (Ninguno/ Embarazada/Lactando /Menopausia)
Eating habits data (Datos de hábitos alimentarios)	
Have you noticed that your eating habits change during your menstrual period? (¿Usted ha notado que cambia hábitos alimentarios en el período menstrual?)	Yes/No (Sí/No)
What do you prefer to eat during your period? (¿Qué prefiere comer en tu menstruación?)	Salty/Sweet/Bitter/None (Salado/Dulce/Amargo/Ninguno en particular)
How many liters of water do you drink a day? (¿Cuántos litros toma de agua simple al día?)	Liters (Litros)
Do you consume alcoholic beverages? (¿Consume usted bebidas alcohólicas ?)	Yes /No (Sí/No)
Do you smoke? (¿ Usted fuma ?)	Yes /No (Sí/No)
Do you consume drugs? (¿Consume usted grogas ?)	Yes /No (Sí/No)
Do you drink coffee? (¿Consume usted café?)	Yes /No (Sí/No)
How often do you eat fruits? (¿Qué tan frecuente consume frutas?)	(Never/ By season/once a month/once or twice a week/ three or five times a week/every day) (Nunca/Por temporada/1 vez por mes/1–2 veces por sem/3–5 veces por sem/Diario)
How often do you eat vegetables? (¿Qué tan frecuente consume verduras?)	(Never/ By season/once a month/once or twice a week/ three or five times a week/ every day) (Nunca/Por temporada/1 vez por mes/1–2 veces por sem/3–5 veces por sem/Diario)
How often do you eat red meat? (¿Qué tan frecuente consume carne roja?)	(Never/ By season/once a month/once or twice a week/ three or five times a week/ every day) (Nunca/Por temporada/1 vez por mes/1–2 veces por sem/3–5 veces por sem/Diario)
How often do you eat fish? (¿Qué tan frecuente consume pescado?)	(Never/ By season/once a month/once or twice a week/ three or five times a week/ every day) (Nunca/Por temporada/1 vez por mes/1–2 veces por sem/3–5 veces por sem/Diario)
How often do you eat chicken? (¿Qué tan frecuente consume pollo?)	(Never/ By season/once a month/once or twice a week/ three or five times a week/ every day) (Nunca/Por temporada/1 vez por mes/1–2 veces por sem/3–5 veces por sem/Diario)
How often do you consume milk? (¿Qué tan frecuente consume leche?)	(Never/ By season/once a month/once or twice a week/ three or five times a week/ every day) (Nunca/Por temporada/1 vez por mes/1–2 veces por sem/3–5 veces por sem/Diario)
How often do you consume eggs? (¿Qué tan frecuente consume huevo?)	(Never/ By season/once a month/once or twice a week/ three or five times a week/ every day) (Nunca/Por temporada/1 vez por mes/1–2 veces por sem/3–5 veces por sem/Diario)
How often do you eat tortilla? (¿Qué tan frecuente consume tortilla?)	(Never/ By season/once a month/once or twice a week/ three or five times a week/ every day) (Nunca/Por temporada/1 vez por mes/1–2 veces por sem/3–5 veces por sem/Diario)
How often do you eat sweet bread? (¿Qué tan frecuente consume pan dulce?)	(Never/By season/once a month/once or twice a week/three or five times a week/ every day) (Nunca/Por temporada/1 vez por mes/1–2 veces por sem/3–5 veces por sem/Diario)
How often do you consume oil? (¿Qué tan frecuente consume aceite?)	(Never/ By season/once a month/once or twice a week/ three or five times a week/ every day) (Nunca/Por temporada/1 vez por mes/1–2 veces por sem/3–5 veces por sem/Diario)
How often do you consume butter? How often do you consume lard? (¿Qué tan frecuente consume manteca?)	(Never/ By season/once a month/once or twice a week/ three or five times a week/ every day) (Nunca/Por temporada/1 vez por mes/1–2 veces por sem/3–5 veces por sem/Diario)
How often do you consume nuts, almonds, seeds, peanuts? (¿Qué tan frecuente consume nueces, almendras, semillas, cacahuates?)	(Never/ By season/once a month/once or twice a week/ three or five times a week/every day) (Nunca/Por temporada/1 vez por mes/1–2 veces por sem/3–5 veces por sem/Diario)
How often do you consume sugar? (¿Qué tan frecuente consume azúcar?)	(Never/ By season/once a month/once or twice a week/three or five times a week/ every day) (Nunca/Por temporada/1 vez por mes/1–2 veces por sem/3–5 veces por sem/Diario)
How often do you eat sweets? (¿Qué tan frecuente consume dulces?)	(Never/ By season/once a month/once or twice a week/three or five times a week/ every day) (Nunca/Por temporada/1 vez por mes/1–2 veces por sem/3–5 veces por sem/Diario)
How often do you consume soda? (¿Qué tan frecuente consume refresco?)	(Never/ By season/once a month/once or twice a week/three or five times a week/ every day) (Nunca/Por temporada/1 vez por mes/1–2 veces por sem/3–5 veces por sem/Diario)
How often do you drink beer? (¿Qué tan frecuente consume cerveza?)	(Never/ By season/once a month/once or twice a week/ three or five times a week/ every day) (Nunca/Por temporada/1 vez por mes/1–2 veces por sem/3–5 veces por sem/Diario)
How often do you consume wine? (¿Qué tan frecuente consume vino?)	(Never/ By season/once a month/once or twice a week/three or five times a week/ every day) (Nunca/Por temporada/1 vez por mes/1–2 veces por sem/3–5 veces por sem/Diario)
How often do you consume Vodka, rum, tequila, etc.? (¿Qué tan frecuente consume Vodka, ron, tequila, etc.?)	(Never/By season/once a month/once or twice a week/three or five times a week/ every day) (Nunca/Por temporada/1 vez por mes/1–2 veces por sem/3–5 veces por sem/Diario)
How often do you consume chocolate? (¿Qué tan frecuente consume chocolate?)	(Never/ By season/once a month/once or twice a week/three or five times a week/ every day) (Nunca/Por temporada/1 vez por mes/1–2 veces por sem/3–5 veces por sem/Diario)
How often do you consume ice cream? (¿Qué tan frecuente consume helado?)	(Never/ By season/once a month/once or twice a week/three or five times a week/ every day) (Nunca/Por temporada/1 vez por mes/1–2 veces por sem/3–5 veces por sem/Diario)
Physical activity data (Datos de actividad física)	
How long does it last to exercise? (¿Cuánto dura haciendo ejercicio?)	I don’t do it/Less than 20 min/Half an hour/More than 1 h (No hago/Menos de 20 min/Media hora/Más de 1 hora)
Do you exercise during menstruation? (¿Durante la menstruación realiza ejercicio?)	Yes /No (Sí/No)
Sleep habits data (Datos de hábitos de sueño)	
In the last 3 months, how long do you usually sleep? (En los últimos 3 meses, ¿Cuánto tiempo duermes generalmente?)	Less than 6 h/From 6 to 9 h/More than 9 h (Menos de 6 horas/De 6 a 9 horas/Más de 9 horas)
How long do you sleep during your menstruation? (¿Cuánto tiempo duermes durante tu menstruación?)	Less than 6 h/From 6 to 9 h/More than 9 h (Menos de 6 horas/De 6 a 9 horas/Más de 9 horas)
